# Supplementary material for: YOLOv8n-BWG-enhanced drone smoke detection: Advancing environmental monitoring efficiency
Source: PLoS One. 2025 May 19;20(5):e0322448. doi: 10.1371/journal.pone.0322448 (PMC12088044; doi:10.1371/journal.pone.0322448)
Supplement: Attachment — Submitted filename: Response to Reviewers.docx [file pone.0322448.s001.docx]

**Response to Reviewers**

Thank you for your valuable comments and suggestions on our manuscript. We have carefully considered each of your remarks and have made the necessary revisions to improve the quality and clarity of our work. Below, please find our detailed responses to each of your comments, along with the specific changes we have implemented in the manuscript.We appreciate your time and effort in reviewing our work.

**Reviewer #1:**

1. **Although the process of collecting, labelling, and enhancing the dataset is described, the division of the dataset in the experiment is not clearly stated. It is suggested to add the division ratio of the dataset.**

Response: Thank you for your suggestion. We have added the dataset splitting ratio in Section 3.1 Dataset Construction. The dataset is split into training, validation, and test sets in the ratio of 7:2:1.

The dataset's limited diversity poses a challenge, as training models could potentially misinterpret noise or extraneous features as relevant characteristics. To mitigate this, the study integrates online data augmentation techniques, such as the introduction of random noise, brightness adjustments, and scaling. These methods produce varied, albeit similar, data in each training cycle, thereby enhancing the model's learning of smoke attributes, its generalization capabilities, and overall accuracy.The augmented dataset was divided into training, validation, and test sets in a 7:2:1 ratio.The augmented images are shown in Figure 8.

2. **The experimental environment and parameter settings provide a detailed description of the hyperparameters required for training, but there is no clear description of the parameter tuning process. Please provide a description of how the optimal parameters were selected. In addition, measures to ensure model stability (e.g., setting random seeds) are missing from the experimental description.**

Response: Thank you for your suggestions. In the experiment, most of the hyperparameters were set according to the recommended values in the official documentation. For some key hyperparameters such as the number of training epochs and batch size, we adopted the following strategies for selection: The number of training epochs was determined by observing the trend of the loss value. During the training process, if the loss value tended to stabilize after a certain number of epochs, we considered that the model had been fully trained and could stop training. As for the batch size, it was adjusted according to the GPU memory of the machine to ensure that no errors occurred due to insufficient GPU memory during the training process.

In addition, in the experiment, we chose a learning rate of 0.01 and adopted the Stochastic Gradient Descent (SGD) algorithm for parameter optimization. The SGD algorithm gradually updates the model parameters by calculating the gradient of each small batch of data, thereby optimizing the loss function. We chose 0.01 as the learning rate because it showed a good convergence speed in preliminary experiments and could effectively train the model within a reasonable time. To ensure a smooth training process and convergence to the optimal solution, we would continuously adjust the learning rate according to the loss changes during training.

To control model complexity and prevent overfitting, we set the weight decay coefficient to 5×10⁻⁵. Weight decay is a regularization technique that aims to prevent the model from overfitting to the training data by penalizing large weight values. By introducing this regularization term, we hope to improve the generalization ability of the model while avoiding the model relying too much on the noise in the training data.

To ensure the stability of the model and improve the reproducibility of the experiment, we fixed the random seed to 42 in all experiments. This can ensure the consistency of random initialization and data partitioning during the training process each time, thus reducing experimental fluctuations caused by these factors.

3. **Parameters can be used to measure the lightweight performance of a model, but there is a lack of descriptions and comparisons of Parameters in the comparison experiments of different models in Table 3. Please add the Parameters metrics in Table 3 and add relevant descriptions in the text.**

Response: Thank you for your suggestions. We have added a column for the model parameters in Table 3 and provided corresponding descriptions.

The specific modifications are as follows:

| Table 3 PERFORMANCE COMPARISON OF DIFFERENT MODELS | | | | | | | |
| --- | --- | --- | --- | --- | --- | --- | --- |
| **Model** | P/% | R/% | F1/% | mAP0.5/% | FPS(f/s) | FLOPs/G | Parameter/M |
| Faster RCNN | 82.3 | 85.7 | 80.1 | 83.1 | 2.1 | 135 | 69.7 |
| SSD | 78.2 | 79.3 | 76.3 | 79.1 | 28 | 29.2 | 18.7 |
| YOLOv4-Tiny | 85.7 | 84.2 | 82.1 | 85.5 | 30.3 | 21.7 | 10.4 |
| YOLOv5s | 90.1 | 87.5 | 85.6 | 89.3 | 35.6 | 16.4 | 7.2 |
| YOLOv7-Tiny | 87.3 | 88.5 | 86.2 | 88.5 | 39.1 | 9.8 | 6.2 |
| YOLOv10n | 90.1 | 91.4 | 88.4 | 90.6 | 47.3 | 6.9 | 3.18 |
| YOLOv11n | 91.3 | 92.0 | 89.9 | 91.2 | 49.5 | 6.7 | 2.69 |
| YOLOv8n-BGW | **93.5** | **94.6** | **92.8** | **94.3** | **55.2** | **6.3** | **2.32** |

Among all models, the floating-point operations (FLOPs) of Faster R-CNN stand at 135G, with a parameter size of 69.7M. The FLOPs and parameter size of the Faster R-CNN model are too large to be embedded in drones for use. The single-stage detection algorithm SSD model has lower accuracy and relatively high FLOPs and parameters, making it unsuitable for meeting the requirements of drone-based chimney smoke detection. The YOLOv4-Tiny and YOLOv5s models have FLOPs of 21.7G and 16.4M, respectively, and parameter sizes of 10.4G and 7.2M. This indicates that the FLOPs and parameter sizes of the YOLOv4-Tiny and YOLOv5s models are too large to meet the requirements for drone deployment. The YOLOv7-Tiny model has higher accuracy but slower detection speed, which is not conducive to mobile deployment on drones.

Although the YOLOv10n and YOLOv11n models have relatively high accuracy, they are slightly inferior in accuracy compared to the proposed YOLOv8n-BGW model, which performs the best among all models, achieving an accuracy of 94.3%. Additionally, the YOLOv8n-BGW model excels in computational performance, with the lowest FLOPs and parameter size of only 6.3G and 2.32M, respectively. This shows that the YOLOv8n-BWG model meets the requirements for both accuracy and complexity in chimney smoke detection. Moreover, the YOLOv8n-BGW model achieves 55.2 frames per second (FPS), meeting the requirements for mobile deployment on drones. In summary, the proposed YOLOv8n-BGW model exhibits the best performance among all models, can be effectively embedded in drones, and meets the accuracy requirements for detection.

4. **The conclusion is a bit too simple, and it is suggested to add what problems are solved by introducing BC2f and other modules.**

Response: Thank you for your suggestions. We have supplemented the problems solved by modules such as BC2f in the conclusion to make the conclusion more detailed.

The specific modifications are as follows:

Effective smoke monitoring can effectively manage and reduce the adverse impacts of industrial emissions on the environment and human health, providing substantial support for air quality improvement and sustainable development. To consider embedding the model into UAV devices, based on YOLOv8n, the BC2f structure, WIoUv2 function, and GSConv convolution were integrated into the YOLOv8n network, resulting in the improved model called YOLOv8n-BWG.

In this study, the BC2f structure effectively reduced computational and storage consumption through an adaptive query mechanism while enhancing the model's perception of input content, focusing on smoke targets, suppressing ineffective features and noise, thereby reducing false detection rates. The WIoUv2 function and dynamic sample allocation strategy re-optimized the loss function, significantly improving the model's focus on small targets and reducing the miss rate of small target smoke. The lightweight GSConv convolution reduced the number of parameters while maintaining high accuracy, effectively lowering model complexity while ensuring computational efficiency.

The results show that on a custom dataset, compared to the YOLOv8n model, the YOLOv8n-BWG model increased the average recognition precision mAP by 4.2%, improved recognition speed by 21.3% per second, reduced FLOPs by 28.9%, and reduced the number of parameters by 26.3%. Through final detection results, it was found that the YOLOv8n-BWG model had higher detection accuracy, faster detection speed, and effectively reduced the miss rate of small targets compared to the YOLOv8n model. This paper also uses a binocular stereo vision system to calculate the exact location of smoke areas, providing more comprehensive and reliable environmental sensing data.

Currently, the YOLOv8n-BWG model achieves a detection speed of 55.2 frames on computer devices and only 13.2 frames on UAV edge devices. Therefore, there is room for optimizing detection speed on edge devices. Future plans include accelerating the model and improving detection speed through model pruning and deploying TensorRT.

**5. There are some awkward sentences in the text, such as ‘The single-stage SSD model, despite lower accuracy, does not meet chimney smoke’ should be The single-stage SSD model does not meet the requirements for chimney exhaust due to its low accuracy.’ Please check the grammar of the manuscript for clarity.**

Response: Thank you for your suggestion. We have revised this sentence as follows: "The single-stage detection algorithm SSD model has lower accuracy and relatively high FLOPs and parameters, making it unsuitable for meeting the requirements of drone-based chimney smoke detection." Moreover, we have checked all sentences in the entire article and modified those with unclear logic or incoherence.

**Reviewer #2:**

(1) **Why not use higher version of YOLO, such as YOLOv10?**

Response: Thank you for your suggestions. Currently, the latest version of YOLO is YOLOv11, which was released on September 30, 2024. YOLOv10 was released in May 2024, and YOLOv8 was released on January 10, 2023. However, the improvements of YOLOv11 and YOLOv10 are based on the YOLOv8 model. Therefore, they share many similarities with YOLOv8 in terms of architecture and implementation, with only a few modules being changed. Secondly, we added the YOLOv10n and YOLOv11n models to Table 3 for comparison. The results show that the proposed YOLOv8n-BGW model still has obvious advantages.

The specific modifications are as follows:

| Table 3 PERFORMANCE COMPARISON OF DIFFERENT MODELS | | | | | | | |
| --- | --- | --- | --- | --- | --- | --- | --- |
| **Model** | P/% | R/% | F1/% | mAP0.5/% | FPS(f/s) | FLOPs/G | Parameter/M |
| Faster RCNN | 82.3 | 85.7 | 80.1 | 83.1 | 2.1 | 135 | 69.7 |
| SSD | 78.2 | 79.3 | 76.3 | 79.1 | 28 | 29.2 | 18.7 |
| YOLOv4-Tiny | 85.7 | 84.2 | 82.1 | 85.5 | 30.3 | 21.7 | 10.4 |
| YOLOv5s | 90.1 | 87.5 | 85.6 | 89.3 | 35.6 | 16.4 | 7.2 |
| YOLOv7-Tiny | 87.3 | 88.5 | 86.2 | 88.5 | 39.1 | 9.8 | 6.2 |
| YOLOv10n | 90.1 | 91.4 | 88.4 | 90.6 | 47.3 | 6.9 | 3.18 |
| YOLOv11n | 91.3 | 92.0 | 89.9 | 91.2 | 49.5 | 6.7 | 2.69 |
| YOLOv8n-BGW | **93.5** | **94.6** | **92.8** | **94.3** | **55.2** | **6.3** | **2.32** |

Among all models, the floating-point operations (FLOPs) of Faster R-CNN stand at 135G, with a parameter size of 69.7M. The FLOPs and parameter size of the Faster R-CNN model are too large to be embedded in drones for use. The single-stage detection algorithm SSD model has lower accuracy and relatively high FLOPs and parameters, making it unsuitable for meeting the requirements of drone-based chimney smoke detection. The YOLOv4-Tiny and YOLOv5s models have FLOPs of 21.7G and 16.4M, respectively, and parameter sizes of 10.4G and 7.2M. This indicates that the FLOPs and parameter sizes of the YOLOv4-Tiny and YOLOv5s models are too large to meet the requirements for drone deployment. The YOLOv7-Tiny model has higher accuracy but slower detection speed, which is not conducive to mobile deployment on drones.

Although the YOLOv10n and YOLOv11n models have relatively high accuracy, they are slightly inferior in accuracy compared to the proposed YOLOv8n-BGW model, which performs the best among all models, achieving an accuracy of 94.3%. Additionally, the YOLOv8n-BGW model excels in computational performance, with the lowest FLOPs and parameter size of only 6.3G and 2.32M, respectively. This shows that the YOLOv8n-BWG model meets the requirements for both accuracy and complexity in chimney smoke detection. Moreover, the YOLOv8n-BGW model achieves 55.2 frames per second (FPS), meeting the requirements for mobile deployment on drones. In summary, the proposed YOLOv8n-BGW model exhibits the best performance among all models, can be effectively embedded in drones, and meets the accuracy requirements for detection.

(2) **Some paragraphs are too long and difficult to follow, e.g. Lines 39~67. Please divide them into several short paragraphs to improve the readability.**

Response: Thank you for your suggestion. We have split the content from line 39 to line 67 to improve the readability of the article.

The specific modifications are as follows:

To optimize real-time target detection and enhance compatibility with edge devices, single-stage algorithms are generally favored. These methods approach object detection as a regression challenge, leveraging convolutional neural networks to directly predict bounding box locations and categories. This eliminates the need for candidate region generation and classification steps required in two-stage algorithms, thereby significantly improving detection speed. Such efficiency renders single-stage algorithms ideal for real-time applications and deployment in resource-constrained environments [17].

In specific studies, Yue et al. [18] modified the SSD framework by replacing its backbone with MobileNets, making it more suitable for mobile and embedded applications. However, this modification compromised detection accuracy, rendering it insufficient for smoke detection requirements. Zhao et al. [19] proposed an enhanced Fire-YOLO algorithm that expands feature extraction capabilities across three dimensions. While this improved small smoke target detection, its processing speed remained inadequate for real-time applications. Wang et al. [20] developed Light-YOLOv4, a lightweight model substituting CSPDarknet53 with MobileNetv3, but achieved suboptimal mean average precision (mAP) for smoke detection standards. Xin et al. [21] introduced an improved YOLOv5-based UAV fire smoke detection system utilizing focalNextBlock modules from CFNET to enhance multi-scale information integration and reduce parameters. Nevertheless, this method underperformed in detecting small smoke targets. Yang et al. [22] enhanced YOLOv5 by integrating PConv for drone-based forest fire detection, reducing model parameters and accelerating processing speed, though with compromised detection accuracy. Xu et al. [23] combined YOLOv5 and EfficientDet with an additional verification classifier, achieving higher smoke detection accuracy but resulting in excessive model size and slow inference speed, making it unsuitable for UAV deployment.

To address high computational complexity in UAV detection models, Hao-Ran et al. [24] developed a lightweight YOLOv7-based smoke detection model. While significantly reducing computational costs compared to original YOLOv7, its detection accuracy remained insufficient. Wang et al. [25] proposed YOLO-CSQ, an improved YOLOv8-based UAV fire detection method featuring a Quadrupled-ASFF detection head for weighted feature fusion. Although this enhanced multi-scale detection robustness, the model struggled to distinguish between clouds and smoke, leading to frequent false positives.

(3) **The review of the related works and comparison experiments can be more sufficient. Please carefully read and compare (if applicable) the following papers on SOTA detection. [DOI: 10.1016/j.patcog.2023.109878; 10.3390/s20041010] If the authors cannot employ these methods or compare their method with these methods, at least they could introduce/mention these technologies in related sections to improve the quality of the survey.**

Response: Thank you for your suggestions. We have thoroughly read the two papers and noticed that they made detailed comparisons regarding accuracy metrics. Specifically, they employed common evaluation metrics from the COCO dataset, such as AP (Average Precision), AP50 (AP at 50% IoU), and AP75 (AP at 75% IoU). These metrics are conducive to evaluating the model performance from diverse perspectives and can offer a more comprehensive assessment of the detection capabilities. Consequently, we have incorporated these accuracy metrics into our article.We will add these two papers and use them as references 36 and 37.

Since our article has already referred to comprehensive performance metrics like mAP, P, R, and F1, while AP, AP50, and AP75 more predominantly reflect the impact of the IoU threshold on detection accuracy, we have taken a comprehensive approach to the experiments. In the Loss Function Ablation Experiment and the Ablation Experiment on Optimization Modules, we added comparisons of AP, AP50, and AP75 to better showcase the performance of our method under different IoU thresholds. Considering that this paper deals with a single-class detection problem, based on the formula, it can be confirmed that mAP50 is equivalent to AP50. Thus, we utilized these metrics for evaluation in these experiments.

In the Comparison Experiment of Different Models, as different models are involved, the emphasis is on conducting an all-around comparison of comprehensive performance (mAP, P, R, and F1). Therefore, we did not introduce accuracy - related metrics such as AP, AP50, and AP75. Nevertheless, to more clearly demonstrate the comprehensive performance advantages of the model we proposed, we added a radar chart in this section to more intuitively reflect the differences in various performance metrics among different models.

We believe that these additions can further enhance the quality of the article and provide readers with a more comprehensive and lucid experimental analysis. Thank you again for your valuable comments, and we will fully consider these feedbacks during the revision.

The specific modifications are as follows:

**Loss Function Ablation Experiment**

| **Table 1.** Performance Comparison of Three Loss Functions | | | | | | | | | |
| --- | --- | --- | --- | --- | --- | --- | --- | --- | --- |
| **Model** | P  /% | R  /% | F1  /% | AP/% | AP  50/% | AP  75/% | mAP  0.5/% | FPS  (f/s) | FLOPs  /G |
| **YOLOv8n(CIoU)** | 90.3 | 89.3 | 88.6 | 40.9 | 90.1 | 59.8 | 90.1 | 45.5 | 8.9 |
| **YOLOv8n-WIoUv1** | 91.8 | 92.1 | 89.7 | 42.1 | 91.4 | 63.1 | 91.4 | **45.5** | 8.9 |
| **YOLOv8n-WIoUv2** | **92.5** | **92.7** | **90.5** | **42.7** | **92.1** | **63.8** | **92.1** | 45.3 | **8.9** |
| **YOLOv8n-WIoUv3** | 92.1 | 92.3 | 90.2 | 42.3 | 92.0 | 63.3 | 92.0 | 45.1 | 8.9 |

From Table 1, it can be observed that using the WIoU loss function has almost no impact on model size or inference speed. When adopting the WIoUv2 loss function, all metrics achieve optimal performance: AP increases to 42.7% (a 1.8% improvement over CIoU), AP50 reaches 92.1% (+2.0%), AP75 rises to 63.8% (+4.0%), and mAP0.5 also improves to 92.1% (+2.0%). The monotonic focusing mechanism in WIoUv2 enhances model performance by prioritizing critical regions during training, which accelerates convergence and mitigates gradient vanishing issues. This mechanism proves particularly advantageous for deeper networks, as it effectively guides the model to focus on critical regions while maintaining training stability and efficiency. Therefore, this study employs WIoUv2 with its monotonic focusing mechanism to optimize both bounding box regression and classification losses, better adapting to deeper network architectures and ultimately improving model performance and training efficiency.

**Ablation Experiment on Optimization Modules**

| **Table 2.** Performance Comparison of Ablation Experiments on Optimization Modules | | | | | | | | | | | | | |
| --- | --- | --- | --- | --- | --- | --- | --- | --- | --- | --- | --- | --- | --- |
| **Model** | BC2f | WIoUv2 | GSConv | P  /% | R  /% | F1  /% | AP  /% | AP50  /% | AP75  /% | mAP  0.5/% | FPS  (f/s) | FLOPs  /G | Parameters/M |
| **YOLOv8n** | × | × | × | 90.3 | 89.3 | 88.6 | 40.9 | 90.1 | 59.8 | 90.1 | 45.5 | 8.9 | 3.15 |
| **YOLOv8n-B** | √ | × | × | 92.1 | 93.7 | 91.1 | 42.2 | 93.4 | 64.1 | 93.4 | 41.3 | 9.2 | 3.51 |
| **YOLOv8n-BW** | √ | √ | × | 93.0 | 94.2 | 92.5 | 42.6 | 94.1 | 64.7 | 94.1 | 41.1 | 9.2 | 3.51 |
| **YOLOv8n-BWG** | √ | √ | √ | **93.5** | **94.6** | **92.8** | **43.7** | **94.3** | **64.9** | **94.3** | **55.2** | **6.3** | **2.32** |

An analysis of Table 2 reveals the following:In the YOLOv8n-B model, the BC2f structure designed in this paper is integrated into the backbone network of YOLOv8n. As a result, its F1 score increases by 2.5%, mAP rises by 3.3%, AP improves by 1.3%, AP50 increases by 3.3%, and AP75 goes up by 4.3%. However, the detection speed decreases by 4.2 f/s, the floating-point operations of the model increase by 0.3 G, and the number of parameters (Parameters) increases by 0.36 M. This indicates that integrating the BC2f structure can enhance the network's focus on chimney smoke, effectively suppress invalid features and noise, improve the model's mean average precision, and also enhance the target localization accuracy at a higher IoU threshold (AP75). Meanwhile, it will increase the computational burden and the model size.

The YOLOv8n-BW model upgrades the original CIoU loss function with the WIoU2 function on the basis of the YOLOv8n-B model. It has little impact on the detection speed and model complexity. Instead, the F1 score increases by 1.5% and the mAP rises by 1.3%. In terms of AP, AP improves by 0.4%, AP50 increases by 0.7%, and AP75 goes up by 0.6%. This shows that the WIoUv2 loss function can effectively improve the model's precision at different IoU thresholds by enhancing the attention to small-sample targets, especially excelling in detecting small targets, while having very little influence on speed and complexity.

The YOLOv8n-BWG model introduces the lightweight convolution GSConv to replace the standard convolution on the basis of the YOLOv8n-BW model. It can be observed that the F1 score increases by 0.3% and the mAP rises by 0.2%. In terms of AP, AP improves by 1.1%, AP50 increases by 0.2%, and AP75 goes up by 0.2%. The detection speed increases by 14.1 f/s, the floating-point operations of the model are reduced by 2.9 G, and the number of parameters is decreased by 1.19 M. This indicates that adopting the GSConv convolution can not only ensure the model's precision but also reduce the number of parameters in the model and improve the detection speed.

**Comparison Experiment of Different Models**

To validate the performance of the improved model, this study selected the current mainstream two-stage detection algorithm Faster R-CNN and single-stage detection algorithms SSD, YOLOv4-Tiny, YOLOv5s, YOLOv7-Tiny, YOLOv10n, and YOLOv11n for comparative experiments. Each model used the same hyperparameters, and the results are resented in Table 3.

| Table 3 PERFORMANCE COMPARISON OF DIFFERENT MODELS | | | | | | | |
| --- | --- | --- | --- | --- | --- | --- | --- |
| **Model** | P/% | R/% | F1/% | mAP0.5/% | FPS(f/s) | FLOPs/G | Parameter/M |
| Faster RCNN | 82.3 | 85.7 | 80.1 | 83.1 | 2.1 | 135 | 69.7 |
| SSD | 78.2 | 79.3 | 76.3 | 79.1 | 28 | 29.2 | 18.7 |
| YOLOv4-Tiny | 85.7 | 84.2 | 82.1 | 85.5 | 30.3 | 21.7 | 10.4 |
| YOLOv5s | 90.1 | 87.5 | 85.6 | 89.3 | 35.6 | 16.4 | 7.2 |
| YOLOv7-Tiny | 87.3 | 88.5 | 86.2 | 88.5 | 39.1 | 9.8 | 6.2 |
| YOLOv10n | 90.1 | 91.4 | 88.4 | 90.6 | 47.3 | 6.9 | 3.18 |
| YOLOv11n | 91.3 | 92.0 | 89.9 | 91.2 | 49.5 | 6.7 | 2.69 |
| YOLOv8n-BGW | **93.5** | **94.6** | **92.8** | **94.3** | **55.2** | **6.3** | **2.32** |

Among all models, the floating-point operations (FLOPs) of Faster R-CNN stand at 135G, with a parameter size of 69.7M. The FLOPs and parameter size of the Faster R-CNN model are too large to be embedded in drones for use. The single-stage detection algorithm SSD model has lower accuracy and relatively high FLOPs and parameters, making it unsuitable for meeting the requirements of drone-based chimney smoke detection. The YOLOv4-Tiny and YOLOv5s models have FLOPs of 21.7G and 16.4M, respectively, and parameter sizes of 10.4G and 7.2M. This indicates that the FLOPs and parameter sizes of the YOLOv4-Tiny and YOLOv5s models are too large to meet the requirements for drone deployment. The YOLOv7-Tiny model has higher accuracy but slower detection speed, which is not conducive to mobile deployment on drones.

Although the YOLOv10n and YOLOv11n models have relatively high accuracy, they are slightly inferior in accuracy compared to the proposed YOLOv8n-BGW model, which performs the best among all models, achieving an accuracy of 94.3%. Additionally, the YOLOv8n-BGW model excels in computational performance, with the lowest FLOPs and parameter size of only 6.3G and 2.32M, respectively. This shows that the YOLOv8n-BWG model meets the requirements for both accuracy and complexity in chimney smoke detection. Moreover, the YOLOv8n-BGW model achieves 55.2 frames per second (FPS), meeting the requirements for mobile deployment on drones. In summary, the proposed YOLOv8n-BGW model exhibits the best performance among all models, can be effectively embedded in drones, and meets the accuracy requirements for detection.

To intuitively compare the comprehensive performance of the models, this paper evaluates eight models across multiple metrics, including precision (P), recall (R), F1 score, mAP0.5, average inference time, floating-point operations, and number of parameters. The results are visualized using a radar chart, as shown in Figure 13. Each curve in the radar chart corresponds to one model. The closer the intersection point of the curve with the axis is to the outer edge, the better the model performs in that metric. The larger the area enclosed by the curve, the more superior the overall performance of the model.

**Fig 13.** Radar Chart of Multi-Model Performance Comparison

As can be seen from Figure 13, although some models (such as YOLOv10n and YOLOv11n) also show good performance in terms of lightweight design, the YOLOv8n-BGW model stands out more prominently in comprehensive performance, especially in model accuracy. This advantage makes the YOLOv8n-BGW more competitive in practical applications and better able to meet the demands of high-precision detection.

(4) **Please provide and label the reference indices of the compared methods in the figures and tables, and then the readers can judge whether the compared methods are SOTA.**

Response: Thank you for your suggestion. Since this study uses a self-built dataset, we have provided the metrics of multiple compared methods in the charts and ensured that all methods are trained and tested on the same dataset to guarantee fairness. Additionally, we have updated the latest models, such as YOLOv10n and YOLOv11n.

(5) **Please use bold font to label the best results in all tables.**

Response: Thank you for your suggestion. We have already bolded the best results in all the tables.

**(6)In Figs. 13(a) and 14(a), are three some overlap problems? One smoke or two smokes?**

Response: Thank you for your suggestion. There is no overlap; the figure indeed represents only one chimney. The reason for the appearance of two bounding boxes is due to the confidence threshold setting of the detection algorithm, which causes different parts of the same chimney to be identified as two separate candidate boxes. Therefore, although two boxes are drawn, they point to the same chimney. To avoid confusion, we have adjusted the detection threshold to ensure that only one box is drawn, accurately reflecting the actual situation.
